# Differential Role of Leptin as an Immunomodulator in Controlling Visceral Leishmaniasis in Normal and Leptin-Deficient Mice

**DOI:** 10.4269/ajtmh.15-0804

**Published:** 2016-07-06

**Authors:** Radheshyam Maurya, Parna Bhattacharya, Nevien Ismail, Pradeep K. Dagur, Amritanshu B. Joshi, Kundan Razdan, J. Philip McCoy, Jill Ascher, Ranadhir Dey, Hira L. Nakhasi

**Affiliations:** ^1^ Division of Emerging and Transfusion Transmitted Diseases, Center for Biologics Evaluation and Research, Food and Drug Administration, Silver Spring, Maryland.; ^2^ Department of Animal Biology, School of Life Science, University of Hyderabad, Hyderabad, India.; ^3^ Flow Cytometry Core, National Heart, Lung, and Blood Institute, National Institutes of Health, Bethesda, Maryland.; ^4^ Division of Veterinary Services, Center for Biologics Evaluation and Research, Food and Drug Administration, Silver Spring, Maryland.

## Abstract

Visceral leishmaniasis (VL) is caused by the protozoan parasite *Leishmania donovani*. There are no vaccines and available drugs against leishmaniasis are toxic. Immunomodulators that specifically boost the anti-microbial activities of the immune cells could alleviate several of these limitations. Therefore, finding novel immunomodulators for VL therapy is a pressing need. This study is aimed to evaluate the immunomodulatory role of leptin, an adipocyte-derived hormone capable of regulating the immune response, in *L. donovani*-infected mice. We observed that recombinant leptin treatment reduced splenic parasite burden compared with non-treated infected normal mice. Decrease in parasite burden correlated with an induction of innate immune response in antigen-presenting cells that showed an increase in nitric oxide, enhanced pro-inflammatory cytokine (interferon gamma [IFNγ], interleukin12 [IL]12, and IL1β) response in the splenocytes, indicating host-protecting Th1 response mediated by leptin. Moreover, in infected normal mice, leptin treatment induced IFNγ production from both CD4^+^ and CD8^+^ T cells, compared with non-treated infected mice. Alternatively, leptin-deficient (Ob/Ob) mice had higher splenic and liver parasite burden compared with the infected normal mice. However, leptin treatment failed to reduce the splenic parasite burden and improve a host-protective cytokine response in these mice. In addition, in contrast to dendritic cells (DCs) from a normal mouse, Ob/Ob mouse–derived DCs showed a defect in the induction of innate immune response on *Leishmania* infection that could not be reversed by leptin treatment. Therefore, our findings reveal that leptin has a differential immunomodulatory effect in controlling VL in normal and Ob/Ob mice.

## Introduction

Visceral leishmaniasis (VL) is an insect vector–borne disease caused by the protozoan parasite *Leishmania donovani*. Approximately 200 million people worldwide living in tropical and subtropical regions are under the threat of such infections. There are 500,000 new clinical cases and approximately 60,000 deaths each year due to VL.^1^ VL is a poverty-associated disease, endemic in the poorest regions of the world and is fatal if untreated. The clinical manifestations of VL are composed of enlargement of the spleen, very high fever, and anemia.^2^–^4^ VL is one of the major health problems in poverty-prone regions of India, Nepal, Sudan, Bangladesh, and most Latin American countries.^5^–^7^

In VL (both in murine and human), resolution of infection depends on the induction of cellular immunity along with the production of pro-inflammatory, or Th1, cytokines.^8^–^15^ Specifically, production of interleukin12 (IL12) by antigen-presenting cells and interferon gamma (IFNγ) by T cells are crucial for controlling the parasite growth and development of host-protective immunity.^8^,^16^ In contrast, susceptibility to VL is correlated with the presence of a Th2-type anti-inflammatory response.^17^ *L. donovani* infection stimulates the expression of Th2-associated cytokines, such as IL10, IL4, and IL13 in murine models. IL10 is an anti-inflammatory cytokine and can be produced by B cells, macrophages, dendritic cells (DCs), and T cells. It is well documented that the elevated levels of IL10 in serum as well as increased IL10 mRNA expression in infected tissue are highly correlated with severe VL.^14^,^18^–^20^ In the murine VL model, IL10 inhibits antimicrobial machinery of macrophages by modulating normal signal transduction mechanisms.^21^ In addition, in human VL, it has been reported that the impaired function of cellular immunity correlates with the progression of active disease due to the inhibitory effects of IL10.^22^ In addition of IL10, IL4 has generally been considered as a Th2 cytokine that helps in the proliferation of the Th2 cell population and thus a significant downregulator of Th1 cell response.^23^,^24^ Although IL4 and IL13 are well-characterized Th2 cytokines, their specific roles in VL are still unclear.^25^,^26^

Presently, there are no vaccines available against VL. Toxicity of available drugs, emergence of drug resistant parasites, and coinfection with human immunodeficiency virus make the drug treatment regimen even more complex.^27^ Because dysfunction in the host immune system plays an active role in the progression and subsequent resolution of infection following therapy, use of immunomodulators could be an effective alternative approach. Several immunomodulators have been evaluated in treating VL with varying degrees of success.^8^,^28^ Leptin is a host adipocyte–derived immunomodulator, capable of stimulating a strong pro-inflammatory response in animal models.^29^,^30^ Leptin deficiency causes susceptibility to infection and inflammatory stimuli and is associated with dysregulation of cytokine production.^31^ Leptin has been revealed to affect both the innate and adaptive branches of the immune system.^32^,^33^ Leptin modulates activity of natural killer cells,^34^ macrophages,^35^,^36^ and neutrophils.^37^ It has also been shown to modulate innate immune responses such as macrophage phagocytosis and regulate T cell response.^35^,^38^–^40^

Leptin induces T cell proliferation and Th1 cytokine production along with concomitant suppression of Th2 response.^33^,^35^,^41^,^42^ Leptin regulates thymic homeostasis and induces the production of inflammatory cytokines (IFNγ and tumor necrosis factor alpha[TNFα] production) by polarizing Th1 response and activation of monocytes/macrophage and DCs.^32^,^33^,^35^,^43^,^44^ These findings are further substantiated by findings in bacterial infection disease models. For example, leptin-deficient (Ob/Ob) or leptin receptor–deficient (db/db) mice showed impaired ability to clear or control infection by *Klebsiella pneumoniae*, *Listeria monocytogenes*, and *Mycobacterium tuberculosis*.^45^–^47^ It has been well documented that leptin levels are reduced during malnutrition. Importantly, epidemiological and experimental animal model studies have demonstrated an increased risk of VL in malnourished hosts.^48^ Of note, the levels of leptin during active VL infection in human have not been determined. Based on the Th1 polarizing function of leptin reported in other studies, it has been hypothesized that diminished leptin levels due to malnutrition may lead to impaired cell-mediated immunity and an enhanced Th2 response in human VL.^49^ It has been shown that leptin effectively reduces the *L. donovani* multiplication in mouse macrophages in vitro when used with low doses of miltefosine, a known drug used for the treatment of leishmaniasis.^50^ However, the in vivo effects of leptin treatment in *L. donovani*-infected mice and a detailed mechanism of leptin-mediated immunomodulation in inducing host protection are yet to be ascertained.

Hence, in this study, we have investigated the role of leptin in *Leishmania* pathogenesis in an in vivo normal mouse and in a Ob/Ob mouse.

## MATERIALS AND METHODS

### Animals and parasites.

In the experiments, 9- to 10-week-old female C57Bl/6 wild-type (Wt) mice and congenic C57BL/6-Ob/Ob mice from the Jackson Laboratory (Bar Harbor, ME) were used. All mice were maintained in the FDA/CBER AAALAC-accredited facility under standard environmental conditions for this species. The *L. donovani* parasites maintained in golden Syrian hamsters were used for infection.^51^

### Ethics statement.

The animal protocol for this study has been approved by the Institutional Animal Care and Use Committee at the Center for Biologics Evaluation and Research, U.S. Food and Drug Administration (ASP 1995#26). Further, the animal protocol is in full accordance with “The guide for the care and use of animals” as described in the U.S. Public Health Service policy on Humane Care and Use of Laboratory Animals 2015 (http://grants.nih.gov/grants/olaw/references/phspolicylabanimals.pdf).

### Experimental infection.

The experiments with normal mice and Ob/Ob mice were performed under similar conditions. Mice were ordered from the same vendor; they were housed together, provided the same food and infected mice on same day from the same inoculum of parasites. Mice were infected via tail vein with 1 × 10^7^ stationary phase Wt *L. donovani* parasites. The animals were divided into six groups; normal (Wt) control, Wt infection, Wt infection + leptin, Ob/Ob control, Ob/Ob infection, and Ob/Ob infection + leptin. In each group, four or five mice were used. At 45 days of postinfection, mice were treated with recombinant murine leptin (Invitrogen, Carlsbad, CA).

### Leptin administration.

Alzet Mini-Osmotic Pumps (Model 2002, Alzet Corp., Cupertino, CA) were filled aseptically with 100 μL reconstituted recombinant leptin (Invitrogen) and primed with saline for 6 hours at room temperature. The mice were anesthetized with 2.5% isoflurane or Hypnom^®^ (Janssen pharmaceutical, Beerse, Belgium). Prior to surgery, the mice received a single dose of Buprenorphine SR-LAB^®^ (ZooPharm, Windsor, CO), a long-acting opioid pain medication at a dose of 1 mg/kg. The pumps were implanted intraperitoneally by a skilled surgeon using aseptic technique, delivering leptin or vehicle phosphate-buffered saline (PBS) at a rate of 0.25 μL/hour for 14 days. This corresponded to leptin being infused at a concentration of 10 μg/day for 2 weeks.^52^ Two weeks after implantation, mice were killed and parasite loads were measured from their visceral organs.

### Plasma leptin, glucose, and cholesterol levels.

Plasma leptin levels were determined by the Mouse Leptin ELISA Kit (DY498; R & D System, Minneapolis, MN). Briefly, 96-well enzyme-linked immunosorbent assay (ELISA) plate was coated with capture antibody (Ab) and incubated overnight at room temperature. The plate was washed with wash buffer (0.05% Tween 20 in PBS) and then blocked with diluent buffer (5% Tween 20 in PBS) for 1 hour at room temperature. The plate was washed again and 100 μL/well diluted plasma (1:20) in diluent buffer was added. The plate was incubated at room temperature for 2 hours. The plate was then washed again and 100 μL/well of Streptavidin-HRP (KPL, Gaithesburg, MD) was added followed by incubation for 20 minutes at room temperature. Next, the plate was washed and 100 μL of the substrate/well was added and incubated for 20 minutes at room temperature. Finally, 50 μL/well of the stop solution was added and incubated for 20 minutes at room temperature. Optical density was measured at 450 nm. Plasma glucose and cholesterol levels were measured by an enzymatic colorimetric method as described in reagents provided in the commercial kit (Sigma, St. Louis, MO).

### Parasite burden in spleen and liver.

Parasite burden from spleen and liver of infected mice was measured by serial dilution method as described previously.^53^

### Multiplex analysis; extracellular cytokine secreted by antigen-stimulated splenocytes.

Splenocytes were plated in 24-well culture plates and stimulated with either freeze thaw *L. donovani* antigen (Ag) (80 μg/mL freeze thawed antigen [FTAg]), or without Ag in complete RPMI 1640 medium (ThermoFisher, NY) and cells were incubated at 37°C in 5% CO_2_, with 95% humidity. After 72 hours of incubation, cell supernatants were collected and stored in −80°C for further analysis. MILLIPLEX Mouse Cytokine/chemokine magnetic panel kit from Millipore (Billerica, MA) was used to analyze the cytokines from the culture supernatants.^53^ The staining plate was prepared according to the kit manual and read in a Luminex-100 (Luminex system, Millipore) using Bio-Plex Manager software 5 (Bio-Rad, Hercules, CA). The cytokine levels were measured by using a standard curve of each specific cytokine.

### Nitric oxide quantification.

Splenocytes were cultured in complete RPMI 1640 medium in the presence or absence of FTAg (80 μg/mL) for 72 hours at 37°C. NO (nitrite/nitrate) production was determined from the supernatants of the cultures by the Griess Reaction Kit (Sigma-Aldrich, St. Louis, MO).^10^

### Ab responses.

The IgG-specific Ab responses were measured by conventional ELISA methods.^53^

### Cultivation of bone marrow–derived macrophages.

Bone marrow cells were isolated from the femurs and tibias of normal and Ob/Ob mice. Cells were cultured with RPMI medium containing fetal bovine serum (FBS) or autologous Ob/Ob mice serum (experiments requiring leptin free conditions) along with 20 ng/mL of macrophage colony stimulating factor. Bone marrow–derived macrophages (BMDMs) were cultured and infected with *L. donovani* parasites as described previously.^54^ To measure parasite load in these cultures, a minimum of 300 macrophages were counted. The results are expressed either as percentages of macrophages that were infected by parasites or as the mean number of parasites/infected macrophage.

### Cultivation of bone marrow–derived DCs.

Normal and Ob/Ob mice were killed to excise femurs and tibias. Bone marrow was isolated and cultured with RPMI medium containing FBS or autologous Lep(Ob/Ob) serum containing 20 ng/mL of granulocyte macrophage colony stimulating factor for experiments requiring leptin-free conditions, for 7 days to obtain > 75% purity of CD11c + DCs.

For the expression of co-stimulatory molecules, bone marrow derived dendritic cells (BMDCs) were infected with various groups of opsonized parasites and 6 hours of postinfection, extracellular parasites were washed out and cells were cultured with or without mouse recombinant leptin (1 μg/mL). At 24 hours of postinfection, cells were stained with different antibodies as described previously.^54^ In another set of experiments for cytokine measurements, DCs were infected with the Wt parasites (*LdWT*) and, after 6 hours, cells were washed with culture medium to remove the extracellular parasites. The infected cells were cultured for 24 hours with or without mouse recombinant leptin (1 μg/mL). Culture supernatants were collected at 24 hours postinfection to evaluate cytokine production by ELISA as described previously.^54^

### Intracellular staining and flow cytometry.

Splenocytes were cultured in 24-well plates in complete RPMI 1640 medium at 37°C and stimulated with or without FTAg (80 μg/mL) for the 36 hours.^53^ After incubation, protein transport inhibitor (BD GolgiStop; BD Pharmingen) was added to the wells and the plate was incubated at 37°C for 3–5 hours. After incubation, cells were blocked at 4°C with rat anti-mouse CD16/32 (5 μg/mL) from BD Pharmingen for 20 minutes. Cells were surface stained with anti-mouse CD3 Alexa Fluor@700, anti-mouse CD4 FITC 488, and anti-mouse CD8 APC Cy7 528 Abs (eBioscience) for 30 minutes at 4°C with 1:200 dilution. The cells were then stained with LIVE/DEAD Fixable Aqua (Invitrogen/Molecular Probes) to mark dead cells. Cells were washed with wash buffer and fixed with the Cytofix/Cytoperm Kit (BD Biosciences, San Jose, CA) for 20 minutes at room temperature. Intracellular staining was performed with anti-mouse IFNγ PE-Cy7 and anti-mouse IL10 PE (eBioscience, San Diego, CA) for 30 minutes at 4°C with 1:200 dilutions. Cells were acquired on LSRII Fortessa (BD Biosciences) using DIVA 6.1.2 software. For analysis, cell doublets were removed using width parameter and dead cells were excluded based on staining with the LIVE/DEAD Aqua dye. Lymphocytes were gated according to their light-scattering properties. CD4^+^ and CD8^+^ T cells were identified as CD3+ lymphocyte–gated populations. Intracellular cytokines were measured in CD4^+^ and CD8^+^ T cell–gated population. Single-stain controls were used for proper gating of positive events for designated cytokines.

### Statistical analysis.

Statistical analysis of differences between means of groups was determined by unpaired two-tailed Student's *t* test, using GraphPad Prism 5.0 software (La Jolla, CA). A *P* value < 0.05 was considered as significant, and a *P* value < 0.01 was considered highly significant.

## RESULTS

### Leptin treatment reduces the parasite growth in *L. donovani*-infected mice.

**Figure 1.**
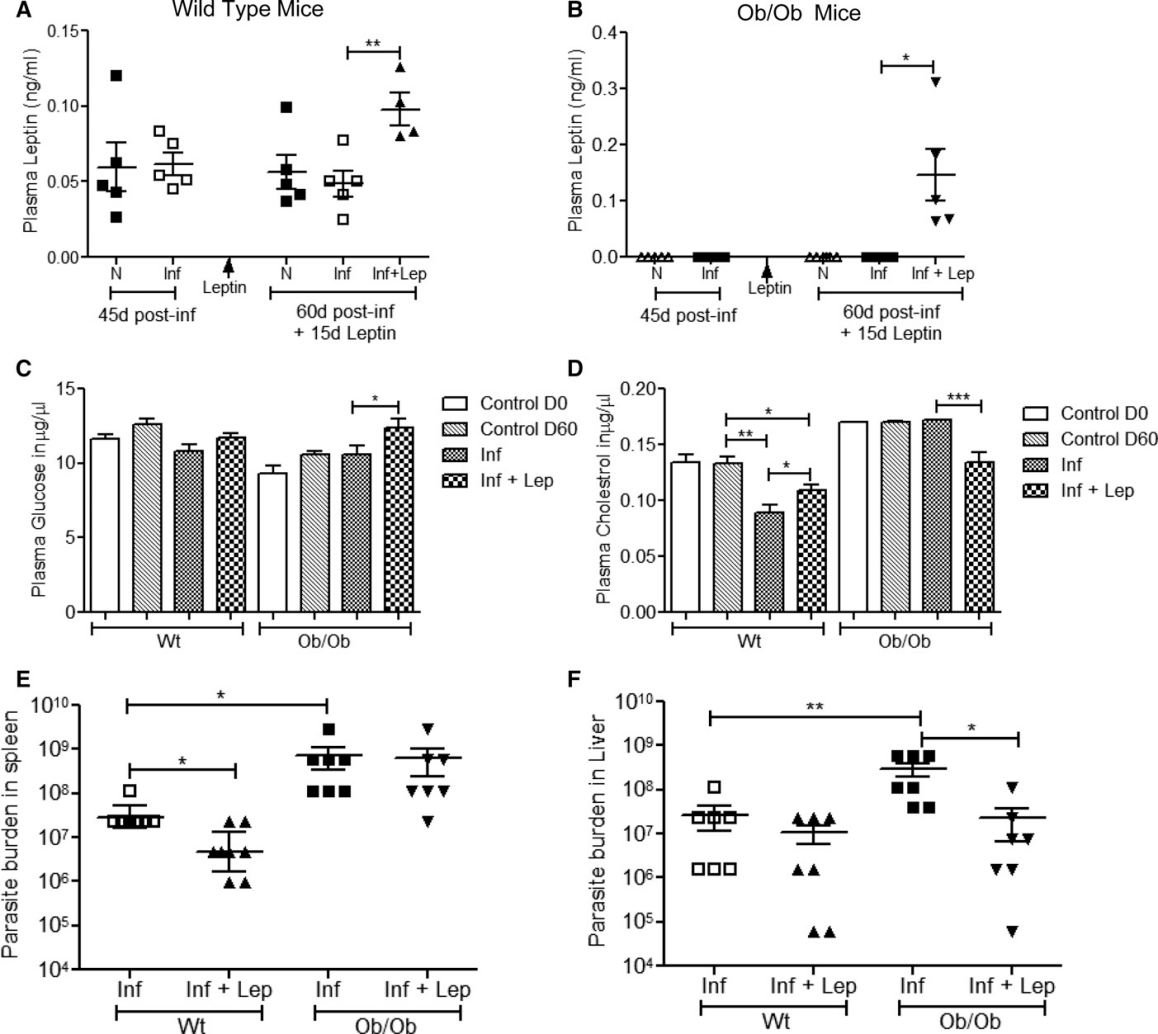
Effect of exogenous leptin administration on plasma leptin, glucose, cholesterol levels, splenic, and liver parasite burden in normal and Ob/Ob mice. On day 0, mice were infected with *Leishmania donovani* by intravenous injection. On day 45 postinfection, leptin delivery pump was implanted in one group of infected mice; 15 days post leptin treatment, mice were euthanized. (**A**) Plasma leptin level of normal mice and (**B**) Ob/Ob mice was measured; naive mice (N), *L. donovani* infected (Inf), and *L. donovani* infected along with leptin treatment (Inf + Lep) at different times postinfection. (**C**) Plasma glucose and (**D**) cholesterol were measured as described in section Materials and Methods. Four to six mice were used in each group, and the experiments were repeated twice. (**E**) Parasite burden from the spleen and (**F**) the liver of different groups of mice at 60 days postinfection was measured and expressed as the geometric mean number of parasites per spleen and liver. Cumulative data of two independent experiments are shown (*N* = 7). * *P* < 0.05, ** *P* < 0.005.

Because leptin has been shown to have an immunomodulatory effect on the infectivity of various pathogens,^38^,^55^ we explored its role in VL pathogenesis. Normal C57Bl/6 mice or Ob/Ob mice were infected for 45 days and then treated with leptin by implanting an intraperitoneal pump for another 15 days. Plasma leptin levels were measured at the end of 45 days postinfection and after 15 days of leptin treatment. In normal mice, similar levels of plasma leptin were observed compared with uninfected normal mice at 45 days of postinfection. At the end of 60 days of infection, mice that were infected and treated with leptin had significantly increased plasma leptin levels compared with untreated infected mice (Figure 1A

). However, as expected, plasma leptin levels were not detectable in uninfected and in 45 days postinfected Ob/Ob mice (Figure 1B). Leptin treatment of infected Ob/Ob mice resulted in loss of body weight (Supplemental Figure 1) along with a significant increase in plasma leptin level compared with untreated Ob/Ob-infected mice (Figure 1B). As leptin plays an important role in regulating metabolism, we measured glucose and cholesterol level from the plasma of these infected and leptin-treated mice. In normal infected or uninfected mice, the plasma glucose level did not change on leptin treatment (Figure 1C). On the other hand, leptin treatment in Ob/Ob-infected mice significantly increased the glucose level compared with non-treated infected mice (Figure 1C). Interestingly, plasma cholesterol level was significantly low in normal infected mice compared with uninfected mice, and leptin treatment partially restored the cholesterol level (Figure 1D). In Ob/Ob mice, plasma cholesterol level was similar in infected or uninfected mice; however, higher than the normal uninfected or infected mice. Leptin treatment significantly reduced the cholesterol level in infected Ob/Ob mice and was similar to normal mice (Figure 1D). To test the effect of leptin treatment on parasite replication in vivo, we determined the splenic and liver parasite burden. In normal infected mice, leptin treatment of 15 days resulted in significant decrease in splenic parasite burden (Figure 1E). Interestingly, the splenic parasite burden was 20-fold higher in Ob/Ob mice compared with normal infected mice (Figure 1E). However, leptin treatment of infected Ob/Ob mice failed to reduce the splenic parasite burden (Figure 1E) unlike infected normal mice. The liver parasite burden of Ob/Ob mice was also significantly higher (11-fold) compared with normal mice (Figure 1F). Although, leptin treatment in normal mice did not significantly reduce liver parasite burden, in Ob/Ob mice, leptin treatment did result in significant reduction of the liver parasite burden (Figure 1F). Overall, these results suggest that leptin treatment has differential effect on controlling splenic and liver parasite burden in both normal and Ob/Ob mice.

### Analysis of pro-inflammatory and anti-inflammatory cytokines secretion in the splenocytes of leptin-treated *L. donovani*-infected normal mice.

**Figure 2.**
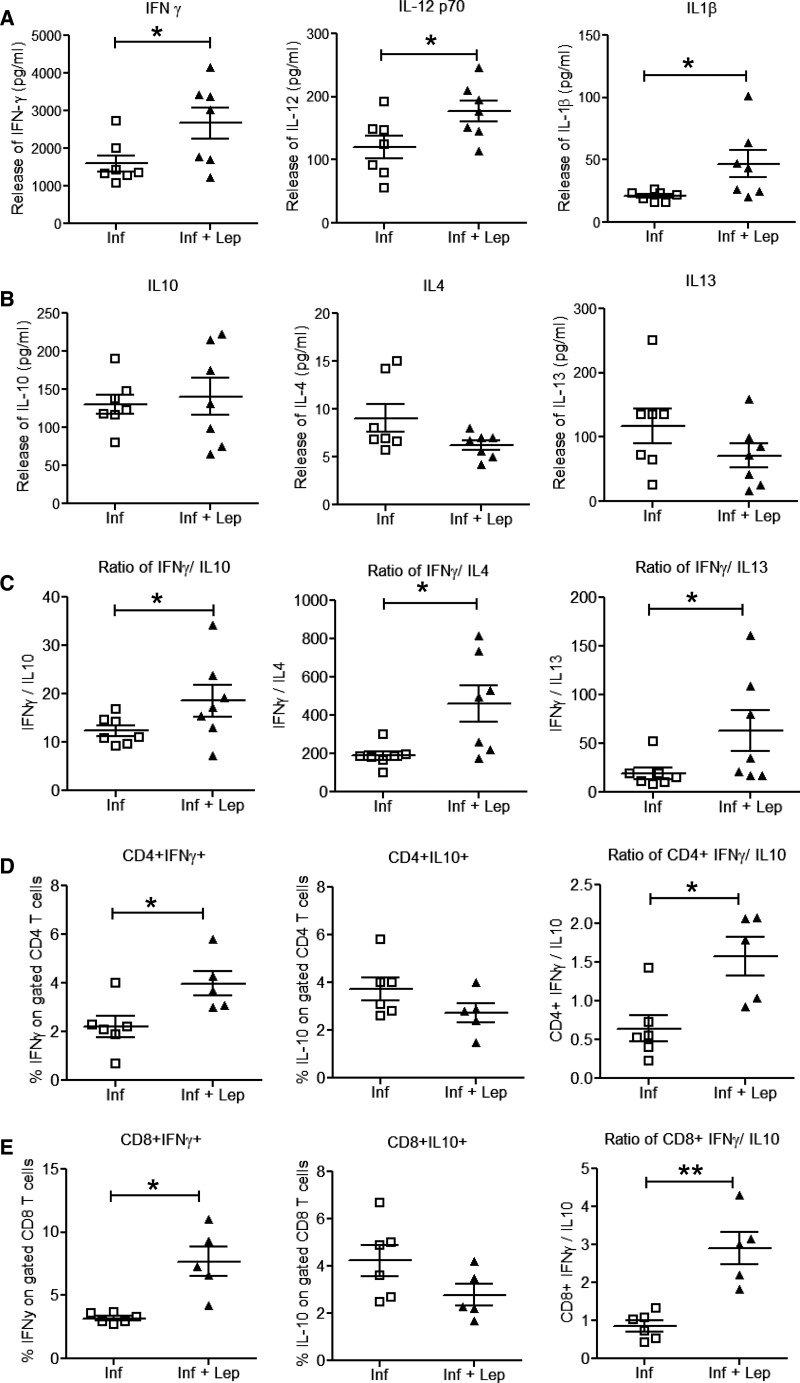
*Leishmania* Ag–stimulated cytokine profiles in splenocyte culture supernatants from leptin-treated and untreated *Leishmania donovani*-infected normal mice. Splenocytes from *L. donovani*-infected and leptin-treated infected normal mice were isolated 60 days after infection, plated aseptically (2 × 10^5^ cells/well), and stimulated with *Leishmania* FTAg for 48 hours. Concentrations of (**A**) pro-inflammatory cytokines interferon (IFN)γ, interleukin (IL)12p70, and IL1β and (**B**) anti-inflammatory cytokines IL10, IL4, and IL13 were measured in culture supernatants using the multiplex mouse cytokine kit as described in the section Material and Methods. (**C**) Ratio of IFNγ/IL10, IFNγ/IL4, and IFNγ/IL13. (**D**, **E**) Percentage of CD4 or CD8 T cells expressing IFNγ and IL10 and the ratio of IFNγ/IL10 are indicated, respectively. Stained cells were analyzed by flow cytometry. Cumulative data of two independent experiments are shown (*N* = 7). * *P* < 0.05, ** *P* < 0.005.

Leptin activates pro-inflammatory cells promoting a Th1 immune response.^42^ Hence, to characterize the immune response induced by leptin treatment during *L. donovani* infection in mice, we analyzed *L. donovani* Ag–specific cytokine secretion by splenocytes from untreated *L. donovani*-infected and leptin-treated infected mice at 60 days after infection. Splenocytes from leptin-treated infected mice produced significantly more Th1-associated cytokines (IFNγ, IL12p70, and IL1β) compared with untreated infected mice (Figure 2A

). Interestingly, leptin-treated infected mice produced similar level of Th2-associated cytokines (IL10, IL4, and IL13) like that of untreated infected mice (Figure 2B). As the balance of Th1/Th2 cytokines determines the fate of VL progression, we also determined ratios of these cytokines. Overall, the ratios of Th1/Th2 cytokines (IFNγ/IL10, IFNγ/IL4, IFNγ/IL13) were significantly higher in splenocytes from leptin-treated infected mice splenocytes compared with untreated infected mice (Figure 2C). Because both CD4^+^ and CD8^+^ T cells are crucial in controlling disease progression during VL, we measured cytokine secretion from splenocyte-derived CD4^+^ and CD8^+^ T cells in response to *L. donovani* Ags. Percentages of IFNγ-producing CD4^+^ (Figure 2D) and CD8^+^ T (Figure 2E) cells were significantly higher in leptin-treated infected mice compared with untreated infected mice after 60 days of infection. Although there were no significant differences in IL10^+^ cells in either CD4^+^ (Figure 2D) or CD8^+^ T (Figure 2E) cells among these two groups, the ratios of IFNγ/IL10–producing cells were significantly high in both CD4^+^ and CD8^+^ T cells in leptin-treated infected mice (Figure 2D and E) compared with untreated infected mice. These results indicate that leptin treatment induces a robust host-protective immune response from CD4^+^ and CD8^+^ T cells in the spleen of infected mice.

### Control of infection by leptin treatment is associated with an induction of NO in splenocytes along with higher IgG2a Ab in plasma of *L. donovani*-infected normal mice.

**Figure 3.**
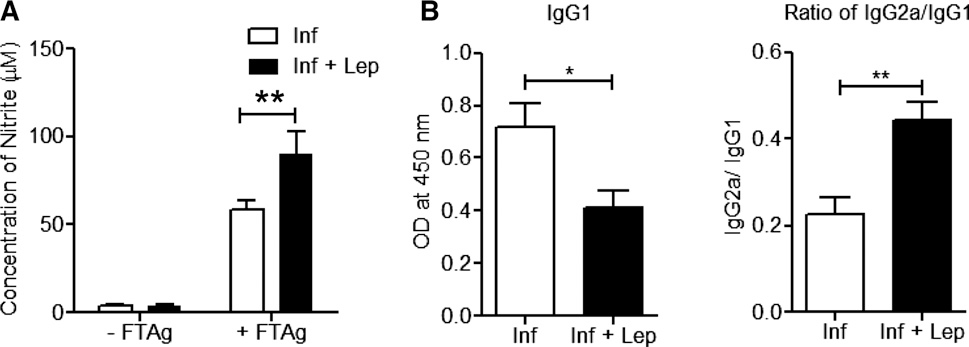
Leptin treatment induces NO (nitrite/nitrate) production by splenocytes from infected mice along with IgG2a response in plasma of normal mice. (**A**) Splenocytes from different groups of infected mice were isolated as described in Figure 2 and the amount of released nitrite in the antigen-stimulated splenocyte supernatants (48 hours) was measured by the Griess reaction. (**B**) Anti-*Leishmania* antibody IgG1 and ratio of IgG2a/IgG1 level from the plasma of leptin treated and untreated *Leishmania donovani*-infected normal mice at 60 days postinfection. The data presented are representative of two independent experiments (*N* = 5 or more) with similar results. Mean and standard error of mean of five or more mice in each group are shown. * *P* < 0.05, ** *P* < 0.005.

Pro-inflammatory cytokines activate macrophages and induce NO production, a key component in *Leishmania* infection control.^51^ Hence, we analyzed the level of NO in the culture supernatants of splenocytes stimulated with soluble *Leishmania* Ags. A significantly higher amount of *Leishmania-*Ag-specific NO production was observed in leptin-treated infected mice compared with untreated infected mice (Figure 3A

). Further, analysis of *Leishmania donovani*-specific immunoglobulin levels showed a decreased level of IgG1 along with a higher IgG2a/IgG1 ratio in leptin-treated infected mice compared with untreated infected mice (Figure 3B). Overall, the results suggest that the higher IgG2a polarized Ab response in plasma of leptin-treated infected mice generates host-protecting Th1 response and induction of NO production in macrophages which in turn leads to reduced parasite burden.

### Analysis of pro- and anti-inflammatory cytokine secretion in splenocytes from infected Ob/Ob mice with or without leptin treatment.

**Figure 4.**
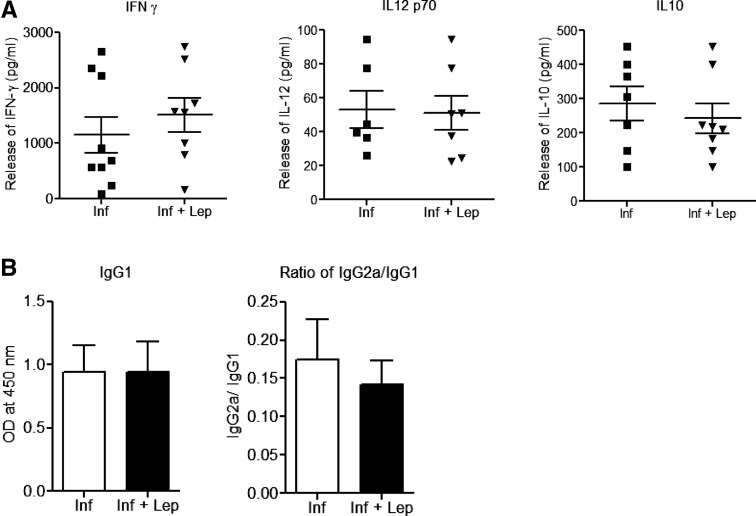
Effect of leptin administration on pro-inflammatory immune response in *Leishmania donovani*-infected Ob/Ob mice. (**A**) Interferon gamma, interleukin (IL)12p70, and IL10 were measured from the *Leishmania* FTAg–stimulated splenocytes culture supernatants using multiplex enzyme-linked immunosorbent assay. (**B**) Anti-*Leishmania* antibody IgG1 and ratio of IgG2a/IgG1 level from the plasma of leptin treated and untreated *L. donovani*-infected mice 60 days postinfection. Cumulative data of two independent experiments are shown (*N* = 7).

To understand the refractoriness of leptin treatment in *L. donovani*-infected Ob/Ob mice, we measured immune response in the splenocytes. In response to soluble *Leishmania* Ags, there were no significant differences in the levels of pro-inflammatory (IFNγ, IL12) and anti-inflammatory (IL10) cytokines in leptin-treated infected Ob/Ob mice splenocytes (Figure 4A

). Moreover, the analysis of plasma from the infected and leptin-treated infected Ob/Ob mice indicated similar levels of IgG1 as well as identical IgG2a/IgG1 ratios, further confirming that leptin treatment did not skew the immune response toward a Th1-type response (Figure 4B). Overall, unlike normal mice, addition of leptin neither induced pro-inflammatory immune response nor reduced the splenic parasite burden in infected Ob/Ob mice. This indicates that leptin deficiency results in a defective immune response in Ob/Ob mice that cannot be rescued by exogenous addition of leptin.

### Effect of leptin on parasite burden in BMDM isolated from normal and Ob/Ob mouse.

**Figure 5.**
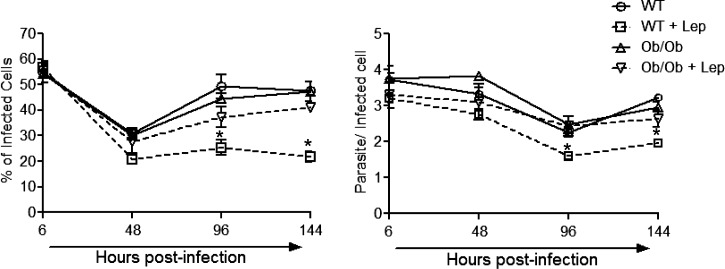
Effect of leptin treatment on *Leishmania donovani* parasite growth in bone marrow–derived macrophages (BMDMs) isolated from normal or Ob/Ob mice. BMDMs isolated from normal and Ob/Ob mice were infected with opsonized *L. donovani* promastigotes (5:1, parasite to macrophage ratio). At 6 hours of postinfection, the extracellular parasites were washed and cells were cultured with or without mouse recombinant leptin (1 μg/mL). At indicated periods of postinfection, intracellular parasite numbers were visualized by Giemsa staining and estimated microscopically. (**A**) Infection efficiency (% of infected cells) and intracellular growth (parasites per infected cell) were recorded at different times postinfection. To measure parasite load in these cultures, a minimum of 300 macrophages were counted. The data represent the means ± standard deviation of three independent experiments. * *P* < 0.05.

To elucidate the mechanism of lack of splenic parasite control and leptin unresponsiveness in Ob/Ob mice, we analyzed the effect of leptin on the parasite burden of *Leishmania*-infected macrophages isolated from Ob/Ob mice and compared it with normal mice. At 6 hours postinfection, there were no significant difference in the percentage of infected cells as well as in the number of parasites per infected cell (Figure 5

) among leptin-treated, untreated normal or Ob/Ob mice. The BMDM cultures from both groups of mice were then subsequently examined at 48, 96, and 144 hours postinfection, and the percentage of infected macrophages and parasite load was calculated. Interestingly, BMDM isolated from normal mice on leptin treatment displayed significantly lower percentage of infected cells and fewer parasites per infected cell compared with untreated infected control macrophages at all the time points postinfection (Figure 5). However, leptin treatment failed to reduce parasite burden in the BMDM isolated from Ob/Ob mice (Figure 5). These results indicate that leptin treatment is potent in reducing the parasite burden in the BMDM of normal mice but not in Ob/Ob mice.

### Analysis of DC function of normal and Ob/Ob mice with and without leptin treatment.

**Figure 6.**
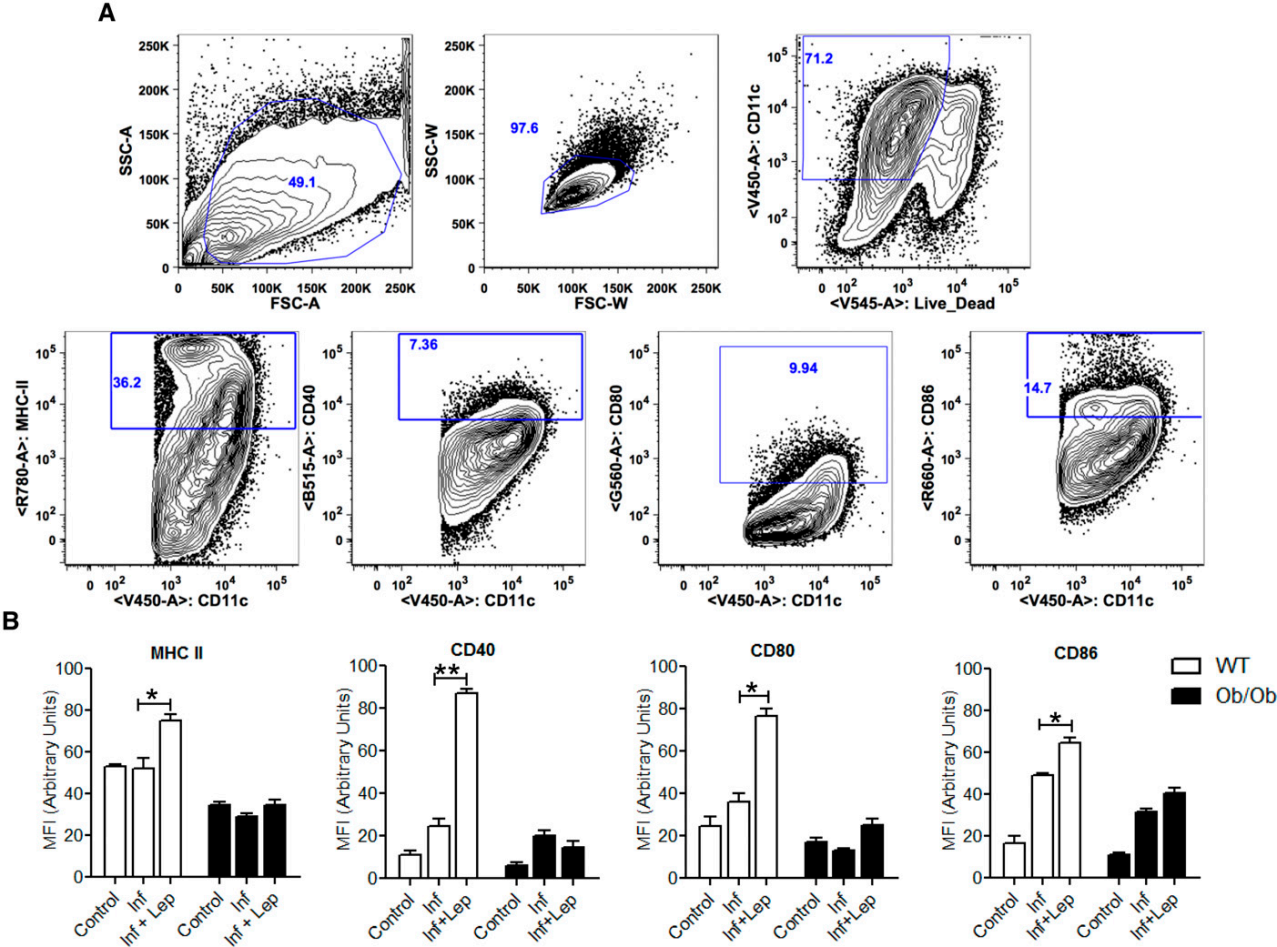
Effect of leptin treatment on the expression of co-stimulatory molecules in infected BMDCs isolated from normal or Ob/Ob mice. BMDCs isolated from normal and Ob/Ob mice were cultured and infected with *Leishmania donovani* promastigotes; 6 hours postinfection, extracellular parasites were washed out and cells were cultured with or without mouse recombinant leptin (1 μg/mL). At 24 hours of postinfection, cells were evaluated for the expression of major histocompatibility complex-II (MHC-II) and the costimulatory molecules CD40, CD80, and CD86 by flow cytometry. (**A**) The dendritic cell population was defined by gating on the CD11c+ cells. (**B**) Bar diagram representing mean fluorescent intensity of the expression of MHC-II, CD40, CD80, and CD86 molecules on the gated CD11c+ cells. The data presented are the means ± standard deviation of two independent experiments. * *P* < 0.05, ** *P* < 0.005.

**Figure 7.**
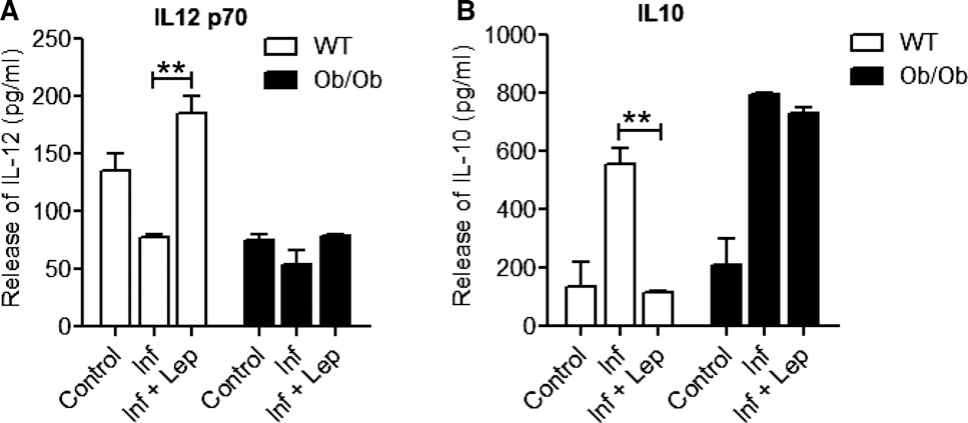
Cytokine analysis of *Leishmania donovani-*infected BMDC culture supernatants in response to leptin treatment. BMDCs isolated from normal and Ob/Ob mice were cultured, infected, and treated with leptin (1 μg/mL). At 24 hours of postinfection, culture supernatants were collected to analyze (**A**) interleukin (IL)12p70 and (**B**) IL10 production by enzyme-linked immunosorbent assay (ELISA) as described in methods. ELISA data are expressed as means ± standard deviation of values from two independent experiments that yielded similar results. ** *P* < 0.005.

Effective clearance of *Leishmania* parasites by macrophages depends on the activation of an appropriate immune response, which is usually initiated by the DCs. Hence, we explored the potential of leptin treatment in modulating the DC function during *Leishmania* infection. We have used an in vitro model of bone marrow–derived DCs isolated from either a normal or an Ob/Ob mouse. The DCs population was defined by gating on the CD11c+ cells by flow cytometry (Figure 6A

) and the expression of costimulatory molecules was measured. Leptin treatment of infected DC from normal mice induced significantly high levels of co-stimulatory molecules (major histocompatibility complex-II [MHC-II], CD40, CD80, and CD86) compared with infected control DCs (Figure 6B) indicating robust antigen presentation due to leptin. However, leptin treatment of infected DCs derived from Ob/Ob mice did not induce the expression of costimulatory molecules compared with untreated infected DCs, thus indicating a defect in antigen presentation in Ob/Ob mice that is not reversible by leptin addition (Figure 6B). Because IL12 produced by DCs plays an important role in the development of Th1 cells during *Leishmania* infection, we examined whether leptin could induce a pro-inflammatory response in DCs in vitro. We observed leptin-treated infected DCs from a normal mouse secreted significantly more IL12 (Figure 7A

) along with a significant reduction in IL10 (Figure 7B) production compared with infected control DCs indicating a skewing toward a pro-inflammatory response. Interestingly, leptin-treated infected DCs from Ob/Ob mice neither induced IL12 (Figure 7A) nor decreased IL10 (Figure 7B) compared with untreated infected control thereby indicating that the DCs from the Ob/Ob mice are unresponsive to leptin treatment. Overall, these data suggest that although leptin treatment enhanced the antigen-presenting function in normal mice DCs, it failed to restore that function in DCs in Ob/Ob mice.

## DISCUSSION

VL is characterized by severe immunosuppression in the host due to increased parasite-driven anti-inflammatory cytokine production. Immunomodulators can be beneficial inactivating the host immune response to overcome the *Leishmania*-induced immunosuppression and restoring immune competence that can lead to resolution of pathology. In the past, several potential immunomodulators have been tested as effective treatments to overcome immunosuppression against both cutaneous and visceral leishmaniasis.^8^,^28^,^56^–^59^ As malnutrition is the primary risk factor that contributes to the development of VL, it has been hypothesized that leptin deficiency during *Leishmania* infection as a result of malnutrition could lead to the impairment of cell-mediated immunity.^49^ However, leptin levels in acute VL patients have not been measured to substantiate this hypothesis. Recently, it has been reported that leptin effectively reduces *L. donovani* multiplication in mouse macrophages in vitro in combination with low doses of miltefosine via augmenting protective immune responses in macrophages suggesting its immunomodulatory role.^50^ However, it is yet to be ascertained whether leptin can protect against *Leishmania*-induced infection in vivo. Hence, in this study, we have evaluated the immunomodulatory role of leptin in regulating the innate and adaptive immune response in *L. donovani*-infected mouse model.

Control of parasitemia in VL is correlated with the induction of Th1 cytokine response.^60^ Leptin has been shown to play a crucial role in activating T lymphocytes toward a pro-inflammatory or Th1 phenotype^61^ via activation of Janus Kinase/signal transducers and activators of transcription signaling pathway.^38^ Leptin stimulates the synthesis of IFNγ, TNFα, and IL12 along with the inhibition of IL10 and IL4.^38^ Maintenance of normal leptin levels is crucial for an appropriate Th1/Th2 balance.^62^ In this study, we found that, leptin treatment could significantly restrict *L. donovani* parasite growth in the spleen of normal mouse. Consistent with this result, we also observed an enhanced Th1 cytokine (IFNγ, IL12, and IL1b) secretion in leptin-treated *L. donovani*-infected mice compared with untreated infected mice. However, leptin-treated infected mice also produced Th2-associated cytokines (IL10, IL4, and IL13) similar to that of untreated infected mice, suggesting a compensatory mechanism to minimize the pro-inflammatory cytokine-mediated tissue injury effect. Interestingly, the ratios of Th1/Th2 cytokines (IFNγ/IL10, IFNγ/IL4, IFNγ/IL13) were significantly higher in leptin-treated infected mice splenocytes compared with untreated infected mice. These results clearly support the idea that leptin is mediating a shift from Th2 to Th1 response in *L. donovani*–infected normal mice, as was observed in infections with other pathogenic agents.^47^,^63^ Furthermore, our results showed that CD4^+^ and CD8^+^ T-cell impairment associated with VL could be rescued by leptin treatment as indicated by the increased percentage of *Leishmania* Ag–specific IFNγ and significantly increased IFNγ/IL10 ratio in both CD4^+^ and CD8^+^ T cells. This protective response of leptin was found to be further mediated by an enhanced production of NO in the splenocytes which in turn leads to significant reduction of parasites in visceral organs. It has also been reported that *Leishmania* infection induces host IgG1, which leads to an increased IL10 secretion, promoting disease exacerbation.^64^ In this study, we observed a decrease in IgG1 and an induction of IgG2a in leptin-treated infected normal mice, which reflects a host-protective Th1 response. These findings demonstrate that leptin treatment could confer protection against *Leishmania* infection in C57Bl/6 mice through an efficient skewing toward a Th1 immune response.

It has been demonstrated earlier that Ob/Ob mice have a dominant Th2-type immune response resulting in increased susceptibility to intracellular infections.^47^ Moreover, in some infections, the immune defects in leptin deficiency are reversed by treatment with recombinant leptin protein. For example, increased susceptibility of Ob/Ob mice toward *K. pneumoniae* was associated with reduced bacterial clearance and defective alveolar macrophage phagocytosis in vitro.^45^ Addition of exogenous leptin enhanced the phagocytosis and increased leukotriene production thereby reversing the course of infection. Similarly, Ob/Ob mice exhibited enhanced lethality and reduced pulmonary bacterial clearance following *Streptococcus pneumoniae* infection. Exogenous leptin administration to Ob/Ob mice improved pulmonary bacterial clearance, reduced bacteremia and killing of *S. pneumoniae* both in vitro and in vivo.^63^ In light of these observations, we examined the immunomodulatory role of leptin during VL. We determined the parasite levels and the immune response in Ob/Ob mice with or without leptin treatment. It is worth mentioning here that leptin has a dual role; as a hormone it is an important regulator of appetites and energy expenditure, whereas as a cytokine it modulates innate and adaptive immune response.^38^ In our study, the plasma leptin levels in leptin-treated Ob/Ob mice were fully restored to the physiological level, as indicated by the reduced body weight as has been also previously reported.^30^ In addition, the restored level of plasma glucose and cholesterol in leptin-treated Ob/Ob mice indicates that hormonal function of leptin is not impaired. However, we observed that Ob/Ob mice, compared with normal mice, exhibited an enhanced parasite burden in both spleen and liver following *L. donovani* infection. Surprisingly, leptin treatment failed to reduce the spleen parasite burden in Ob/Ob mice although liver parasite burden is reduced. The lack of effect of leptin on splenic parasite burden correlated with a lack of induction of pro-inflammatory cytokine response and an absence of skewing toward a Th1 response in the splenocytes. Taken together, our studies suggest that in infected Ob/Ob mice, although the leptin treatment restores the hormonal function, it is unable or inadequate to restore the host-protective immune response. There could be two possible explanations for the unresponsiveness of Ob/Ob mice to leptin treatment during *L. donovani* infection. First, the compromised immune response in Ob/Ob mice is further dampened by virulent *L. donovani* infection, which is not reversible by exogenous leptin treatment. Second, effective dose of leptin and duration of leptin treatment in our study might not be adequate to restore the immune function.

To further investigate the mechanism of leptin function in normal or Ob/Ob mice during *Leishmania* pathogenesis, we extended our study in vitro to determine the innate immune response. Activation of the innate immune response occurs during *Leishmania* infection and can impact the adaptive immune response.^54^ Several studies have shown that leptin modulates immune responses by acting on antigen-presenting cells, macrophages, and DCs.^35^,^41^,^65^ We therefore investigated whether leptin mediates its activity on T cells during *Leishmania* pathogenesis by influencing innate cell function. In particular, leptin enhances the survival of DCs and increases the expression of surface costimulatory molecules such as MHC-II, CD80, and CD86 and consequently increasing antigen presentation capability.^32^ In this study, we found that leptin treatment is potent in reducing the parasite burden in BMDMs from the normal mice but not Ob/Ob mice. Moreover, leptin treatment induced the antigen-presenting function of normal mice DCs infected with *L. donovani* as indicated by induction of co-stimulatory molecules and pro-inflammatory cytokines. However, in infected DCs from Ob/Ob mice, leptin deficiency resulted in defective antigen presentation function, which was not reversed by leptin treatment. Taken together, these results suggest that defective antigenic presentation could underlie a lack of protective immune response in Ob/Ob mice. Further, studies are needed to explore the mechanism of altered immune response in leptin deficiency.

Finally, to our knowledge, this is the first report on the anti-leishmanial role of leptin in an animal model. Our results indicate that leptin has a differential immunomodulatory effect in controlling VL in normal and Ob/Ob mice. This study suggests that leptin can be used as an immunotherapeutic molecule against VL. These findings might help in the development of new therapeutics involving leptin that may prove efficacious against this deadly disease.

## Supplementary Material

Supplemental Figures.
