# Supplementary material for: Differential Role of Leptin as an Immunomodulator in Controlling Visceral Leishmaniasis in Normal and Leptin-Deficient Mice
Source: Am J Trop Med Hyg. 2016 Jul 6;95(1):109–19. doi: 10.4269/ajtmh.15-0804 (PMC4944674; doi:10.4269/ajtmh.15-0804)
Supplement: Supplementary file 1 [file SD2.pdf]

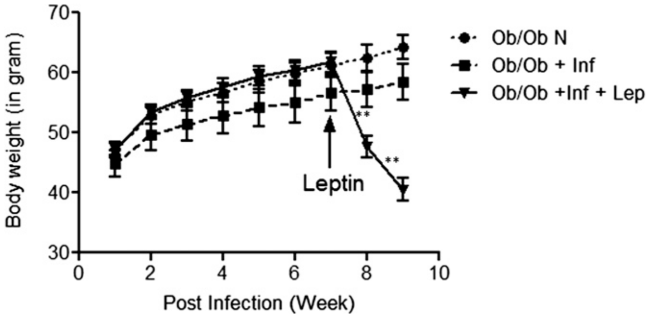

SUPPLEMENTAL FIGURE 1. Body weight change in *Leishmania donovani*-infected Ob/Ob mice after 15 days of leptin treatment. The data presented are the means ± standard deviation of two independent experiments. ** $P < 0.005$.
